# Evaluation of ARID1A as a Potential Biomarker for Predicting Response to Immune Checkpoint Inhibitors in Patients with Endometrial Cancer

**DOI:** 10.3390/cancers16111999

**Published:** 2024-05-24

**Authors:** Hitomi Yamashita, Kentaro Nakayama, Kosuke Kanno, Tomoka Ishibashi, Masako Ishikawa, Kouji Iida, Sultana Razia, Tohru Kiyono, Satoru Kyo

**Affiliations:** 1 Department of Obstetrics and Gynecology, Shimane University School of Medicine, Izumo 693-8501, Japan; meme1103@med.shimane-u.ac.jp (H.Y.); kanno39@med.shimane-u.ac.jp (K.K.); m-ishi@med.shimane-u.ac.jp (M.I.); iida@med.shimane-u.ac.jp (K.I.); 2 Department of Obstetrics and Gynecology, Nagoya City University East Medical Centre, Nagoya 464-8547, Japan; tomoka@med.nagoya-cu.ac.jp; 3 Department of Legal Medicine, Shimane University School of Medicine, Izumo 693-8501, Japan; sultana@med.shimane-u.ac.jp; 4 Project for Prevention of HPV-Related Cancer, Exploratory Oncology Research and Clinical Trial Center (EPOC), National Cancer Center, Kashiwa 277-8577, Japan; tkiyono@east.ncc.go.jp

**Keywords:** endometrial cancer, ARID1A, immune checkpoint inhibitor, immunohistochemistry

## Abstract

**Simple Summary:**

Endometrial cancer is the most common gynecological cancer. However, advanced and recurrent cancers are less sensitive to chemotherapy and have poor prognoses. Therefore, new treatment strategies are being explored. Recently, immune checkpoint inhibitors (ICIs) have been used in treating various cancers. Although AT-rich interaction domain 1A (ARID1A) negativity has been proposed as a new biomarker for immune checkpoint inhibitors, there have been no reports on ARID1A biomarkers in endometrial cancer. Therefore, we investigated whether ARID1A negativity predicts the efficacy of ICIs in treating endometrial cancer. We assessed ARID1A expression and tumor-infiltrating lymphocytes (CD8+) and immune checkpoint molecules (PD-L1/PD-1) using immunostaining and MSI analysis. Throughout our experiment, CD8 and PD-1 expression did not differ significantly between the ARID1A-negative and ARID1A-positive groups. Our findings suggest that ARID1A negativity may not be a suitable biomarker for ICI efficacy in endometrial cancer.

**Abstract:**

Background: AT-rich interaction domain 1A (ARID1A) has been proposed as a new biomarker for predicting response to immune checkpoint inhibitors (ICIs). The predictive value of ARID1A for predicting ICI effectiveness has not been reported for endometrial cancer. Therefore, we investigated whether ARID1A negativity predicts ICI effectiveness for endometrial cancer treatment. Methods: We evaluated ARID1A expression, tumor-infiltrating lymphocytes (CD8+), and immune checkpoint molecules (PD-L1/PD-1) by immunostaining endometrial samples from patients with endometrial cancer. Samples in which any of the four mismatch repair proteins (MLH1, MSH2, MSH6, and PMS2) were determined to be negative via immunostaining were excluded. In the ARID1A-negative group, microsatellite instability (MSI) status was confirmed via MSI analysis. Results: Of the 102 samples investigated, 25 (24.5%) were ARID1A-negative. CD8 and PD-1 expression did not differ significantly between the ARID1A-negative group and the ARID1A-positive group; however, the ARID1A-negative group showed significantly lower PD-L1 expression. Only three samples (14.2%) in the ARID1A-negative group showed high MSI. Sanger sequencing detected three cases of pathological mutation in the MSH2-binding regions. We also established an ARID1A-knockout human ovarian endometriotic epithelial cell line (HMOsisEC7 ARID1A KO), which remained microsatellite-stable after passage. Conclusion: ARID1A negativity is not suitable as a biomarker for ICI effectiveness in treating endometrial cancer.

## 1. Introduction

With the advent of next-generation sequencing (NGS), the rapid genetic analysis of tumor tissues has become possible. As a result, treatments personalized according to genetic mutation are gradually being implemented in clinical practice. Regarding lung cancer, genetic tests (epidermal growth factor receptor [*EGFR*] mutation, anaplastic lymphoma kinase [*ALK*] fusion gene, *ROS1* fusion gene, and *BRAF* V600E mutation) and their respective treatments are covered by insurance, and precision medicine is employed according to the specific genetic mutations observed [1,2,3,4,5,6,7]. However, treatment according to genetic mutations has not yet been established in endometrial cancer treatment. Endometrial cancer is one of the most common gynecologic cancers, and its incidence is gradually increasing [8]. The primary treatment for endometrial cancer is surgery with postoperative adjuvant chemotherapy. However, recurrent and advanced cancers are extremely resistant to chemotherapy and have poor prognoses [9]. Therefore, new therapeutic strategies are required.

The anti-programmed cell death-1 (PD-1) antibody pembrolizumab, which is an immune checkpoint inhibitor (ICI), is used to treat microsatellite instability (MSI)-high solid tumors [10]. Based on the results of the comprehensive genetic analysis of endometrial cancer, The Cancer Genome Atlas Research Network classified endometrial cancer into four molecular phenotypes (polymerase epsilon [POLE]-ultramutated, MSI-hypermutated, copy number high, and copy number low) [11]. The prevalence of MSI-high in endometrial cancer is approximately 30%. Because the proportion of MSI-high tumors is higher in endometrial carcinoma than in other carcinomas, ICIs can be expected to be effective for treating endometrial cancer [12,13].

Recently, it was reported that a deficiency of AT-rich interaction domain 1A (ARID1A) is potentially useful as a biomarker for predicting response to ICIs [14]. *ARID1A* is a tumor suppressor gene that encodes a subunit of the chromatin remodeling complex SWI/SNF [15]. *ARID1A* mutations lead to loss of function, and there are currently no effective therapeutic drugs targeting *ARID1A*. However, *ARID1A* is mutated in many cancers, including ovarian cancer, endometrial cancer, gastric cancer, and bladder cancer [16,17,18,19]. *ARID1A* mutations have been reported to be a poor prognostic factor in various cancers, including ovarian cancer, breast cancer, and gastric cancer [20,21,22]. Therefore, therapeutic drugs targeting *ARID1A* are needed. Shen et al. [14] reported that ARID1A interacts with one of the mismatch repair (MMR) proteins, namely, MSH2. If ARID1A is deficient, MSH2 fails to function and exhibits MSI-high. *ARID1A* mutations are associated with high expression of programmed cell death-ligand 1 (PD-L1) and MSI-high, and ICIs are generally effective in treating *ARID1A*-mutated cancers [23,24]. The relationship between ARID1A deficiency and the efficacy of ICIs in endometrial cancer has not yet been analyzed. Therefore, we investigated the suitability of ARID1A deficiency as a biomarker for predicting response to ICIs in endometrial cancer.

## 2. Materials and Methods

### 2.1. Ethics Statement

This study was conducted in accordance with the ethical standards of national and international guidelines and the Declaration of Helsinki. It was also approved by the Institutional Review Board of Shimane University Hospital (approval number 2004-0381). After approval by the Institutional Review Board, patients’ written consent was obtained to collect tumor specimens.

### 2.2. Tissue Samples

We collected tissue samples from 102 patients with endometrial carcinoma (62 with Grade 1, 32 with Grade 2, and 8 with Grade 3) treated between January 2006 and January 2017 in the Department of Obstetrics and Gynecology of Shimane University Hospital in Japan. The patients were initially treated as follows: total hysterectomy for 39 patients, modified radical hysterectomy for 51, and radical hysterectomy for 9. Three patients underwent chemotherapy and/or radiotherapy without surgery due to complications. Retroperitoneal lymph node dissection was performed in 86 patients. Radiotherapy (whole pelvic irradiation) and/or chemotherapy (using 175 mg/m^2^ of paclitaxel and with a carboplatin area under the curve = 5 mg/mL*min) was performed postoperatively for patients with high recurrence risk (deep myometrial invasion, Grade 2 or 3; lymph node metastasis; or lymphovascular space invasion). The samples were formalin-fixed and converted to paraffin-embedded tissue blocks. The samples were assessed by pathologists after being stained with hematoxylin and eosin. To evaluate the interaction between ARID1A and MSH2, we excluded samples in which one or more of the four MMR proteins (MLH1, MSH2, MSH6, PMS2) were negative. We confirmed MMR protein expression in 102 samples via immunohistochemistry (Supplementary Figure S1).

We used the International Federation of Gynecology and Obstetrics (FIGO) 2014 guidelines for endometrial cancer to stage the tumors [25]. The histological diagnosis of endometrial carcinomas was performed according to the 2014 World Health Organization criteria. The clinical data were collected by retrospective review.

### 2.3. Immunohistochemistry

We evaluated the expression of ARID1A, immune checkpoint molecules (PD-L1 and PD-1), CD8 tumor infiltration, and MMR proteins (MLH1, MSH2, MSH6, and PMS2) using immunohistochemistry.

Formalin-fixed and paraffin-embedded sections (4 μm thick) were immunostained as previously described [26]. We used antibodies against ARID1A (anti-ARID1A antibody sc-32761, mouse monoclonal antibody; Santa Cruz Biotechnology, Dallas, TX, USA), PD-L1 (SP263, Rabbit Monoclonal Primary Antibody; Roche, Basel, Switzerland), PD-1 (NAT105 Mouse Monoclonal Antibody; Roche), CD8 (SP57 Rabbit Monoclonal Primary Antibody; Roche), MutL Protein Homolog 1 (1:50; Dako, Santa Clara, CA, USA), MutS Protein Homolog 2 (1:50; Dako), MutS Protein Homolog 6 (1:50; Dako), and Postmeiotic Segregation Increased 2 (PMS2; 1:40; Dako).

Samples in which no ARID1A expression was found in the nuclei of the tumor cells were evaluated as negative (Supplementary Figure S2). ARID1A’s C-terminal region (1600–1800 amino acids) is essential for its interaction with MSH2 [14].

An anti-ARID1A antibody that binds to 600–1018 amino acids of ARID1A was used for immunohistochemistry. Therefore, it can be inferred that the interaction with MSH2 was also lost in the samples that were determined to be ARID1A negative via immunohistochemistry (Supplementary Figure S3).

The immunostaining of PD-L1, PD-1, and CD8 was performed as previously described [26]. Samples were evaluated as PD-L1-positive if 5% or more of the tumor cells (membrane and cytoplasmic) were stained. Samples were evaluated as PD-1-positive if 5% or more of the tumor-infiltrating lymphocytes were stained. Tumor-infiltrating lymphocyte expression was evaluated in 4 levels (0, undetectable; 1+, low density; 2+, moderate density; and 3+, high density). Samples that stained 2+ or 3+ were evaluated as CD8-positive. Since PD-L1 can be expressed not only in tumor cells but also in immune cells, it can be suspected as being positive in cases with a mixture of tumor cells and immune-related cells. We compared HE staining with PD-1 and PD-L1 immunostaining and carefully observed the cell morphology to clearly distinguish tumor cells from immune cells.

### 2.4. Microsatellite Instability Analysis

MSI analysis was performed to investigate whether samples that were determined ARID1A-negative via immunostaining were MSI-high. DNA was extracted from paraffin-embedded sections of normal tissues and tumor tissues, respectively. MSI analysis was performed using the MSI test (CDx) (BML, Tokyo, Japan) [27], in which a polymerase chain reaction (PCR) with five microsatellite markers (BAT25, BAT26, NR21, MONO27, and NR24) is performed. Samples were assessed as being MSI-high if two or more markers showed length differences between normal tissue and tumor tissue. The remaining samples were assessed as microsatellite-stable (MSS).

### 2.5. Sanger Sequencing

*ARID1A* mutations that are located on the C-terminal side of amino acids 600–1018 are considered ARID1A-positive in immunostaining (The anti-ARID1A antibody used in immunostaining binds to amino acids 600–1018 of ARID1A). Therefore, we performed Sanger sequencing of ARID1A at the regions that bind to MSH2 in samples that were determined ARID1A-positive via immunohistochemistry.

DNA was extracted from endometrial carcinomas and amplified with PCR using primers for exons 18-c, 18-d, 19, and 20a of ARID1A. The primers are shown in Supplementary Figure S4. Sequencing was performed using the ABI BigDye Terminator v3.1 Cycle Sequencing Kit (Applied Biosystems, Foster City, CA, USA). All mutations identified in tumors were evaluated for pathogenic variant by reference to the Catalogue of Somatic Mutations in Cancer (COSMIC).

### 2.6. ARID1A Knockout Human Ovarian Endometriotic Epithelial Cell Line

Epithelial cells derived from ovarian endometriotic cysts were extracted via primary culturing; then, mutant *CDK4* (CDK4^R24C^, an inhibitor-resistant form of CDK4), *Cyclin D1*, and *hTERT* were introduced using lentiviral vectors, and the immortalized cell line HMOsisEC7 was successfully established [28]. Subsequently, we aimed to establish HMOsisEC7 (ARID1A knockout) by using CRISPR-Cas9. HMOsisEC7 was transfected with piggyback vectors, PB-TAC-ERN-3xFlag-hCas9, PB-TKbsd-U6/H1R-ARID1A-gRNA401-394, and PB-TKbsd-U6/H1R-ARID1A-gRNA416-423, and pCAG-PBase-M282V was introduced via electroporation using an NEPA21 instrument (Nepagene, Chiba, Japan). Detailed information on plasmids is provided in the Supplementary Materials [29,30,31]. HMOsisEC7 cells mixed with 2 μg of PB-TAC-ERN-3xFlag-hCas9, 2 μg of PB-TKbsd-U6/H1R-ARID1A-gRNA401-394, 2 μg of PB-TKbsd-U6/H1R-ARID1A-gRNA416-423, 4 μg of pCAG-PBase-M282V, and 0.1 μg of pCMV-EGFP in 100 μL of OptiMEM medium were pulsed using NEPA21. The parameters were as follows: voltage, 175 V; pulse length, 5 ms; pulse interval, 50 ms; number of pulses, 2; decay rate, 10%; polarity + as poring pulse and voltage, 20 V; pulse length, 50 ms; pulse interval, 50 ms; number of pulses, 5; decay rate, 40%; and polarity +/− as the transfer pulse. Subsequently, the mixtures were rapidly transferred to three wells in a six-well plate with the complete culture medium. The cells were cultivated in the presence of 8 μg/mL of Blasticidin S and 100 μg/mL of G418 for 7 days and then treated with 1 μg/mL of doxycycline for 2 weeks. The cells were seeded into three 90 mm dishes at a density of 100 cells/dish, and 24-well-isolated colonies were trypsinized with cloning cylinders and transferred into a 24-well plate. After propagation in a 6-well plate, cellular protein and DNA were extracted from each clone and subjected to Western blot and Sanger sequencing with genomic PCR to confirm the ARID1A status. The genomic DNAs were amplified using PCR with forward primer 5′-GATCAGATGGGCAAGATGAGAC-3′ and reverse primer 5′-GTACCTGTGACCAGGGAGTAAGTAGT-3′. Clones #1, 4, 13, 15, 17, and 19 were confirmed to be ARID1A KO clones.

**Figure 1:**
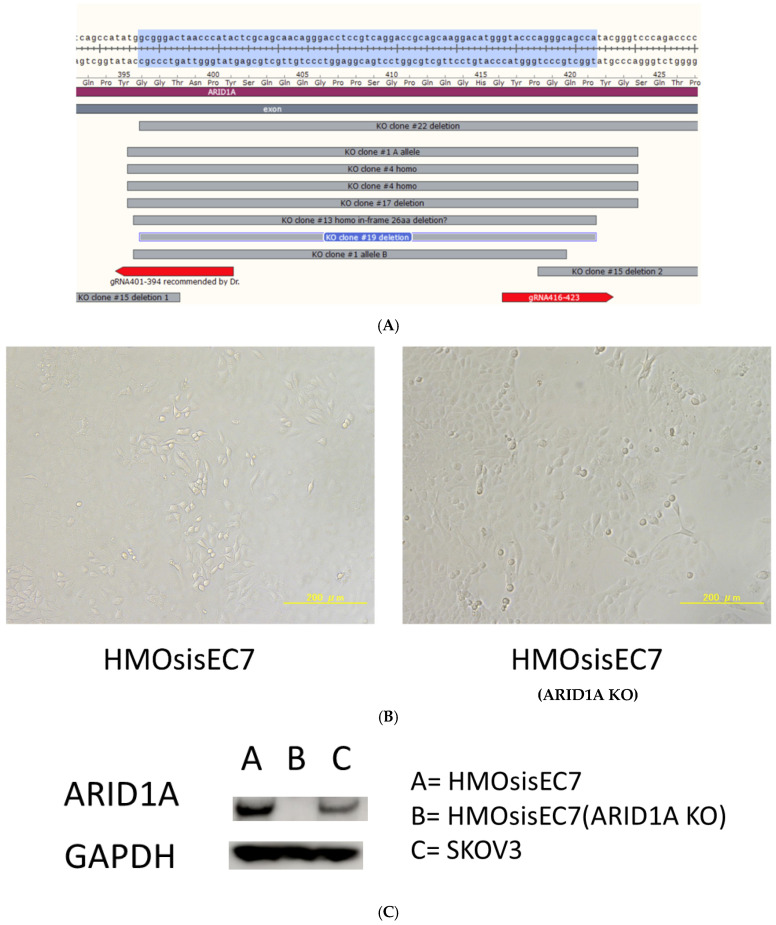
(**A**) Genomic analysis of endometrial cancer samples. Clones #1, 4, 13, 15, 17, and 19 are ARID1A KO clones. We designated clone #19 as HMOsisEC7 (ARID1A KO) and used it for experiments. (**B**) Micrographs of HMOsisEC7 and HMOsisEC7 (ARID1A KO). (**C**) Western blot showing lack of ARID1A expression in HMOsisEC7 (ARID1A KO). The uncropped blots are shown in Figure S5.

Clone #19, which had homologous 86 bp deletion between NT 1185 and 1270 of the ARID1A coding sequence, was further propagated, and the cells were transfected with 10 μg of pCAG-hyperPBase-i7EX via electroporation with NEPA21, followed by selection in the presence of 8 μg/mL of ganciclovir. Ganciclovir-resistant clones #19–30 were propagated and used for further experiments (Figure 1, Supplementary Figure S5). We performed MSI analysis of the *ARID1A*-knockout human ovarian endometriotic epithelial cell line (HMOsisEC7 ARID1A KO).

### 2.7. Statistical Analyses

We performed univariable analysis for progression-free survival (PFS) and overall survival (OS). PFS and OS were calculated between the date of diagnosis and the date of first relapse and last follow-up, respectively. The data are shown as Kaplan–Meier curves. The log-rank test was used to test for the statistical significance of differences in survival between groups. The chi-squared test was used to assess the significance of the association between ARID1A and the expression of CD8, PD-1, and PD-L1. *p*-values < 0.05 were considered statistically significant. The statistical calculations were performed using Statistical Package for the Social Sciences 23.0 (SPSS Inc., Chicago, IL, USA).

## 3. Results

### 3.1. Relationship between ARID1A and Clinicopathological Factors

Immunostaining for ARID1A showed the loss of ARID1A expression in 25 of 102 samples (24.5%). We compared the clinicopathological characteristics of the ARID1A-negative and ARID1A-positive groups. There were no significant differences between the two groups in any of the factors compared. The clinicopathological characteristics of the study participants and their tumors are shown in Table 1.

### 3.2. Relationship between ARID1A and Expression of PD-L1, PD-1 and CD8

**Figure 2:**
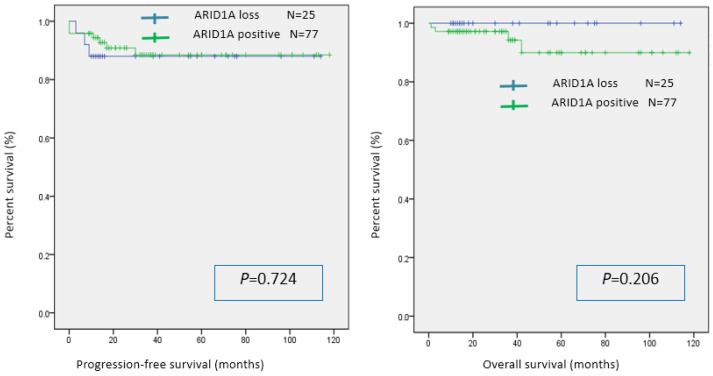
Kaplan–Meier analysis of progression-free survival and overall survival in the ARID1A-positive and ARID1A-negative groups.

The statistical significance of the association between ARID1A expression and immune checkpoint molecules/intratumoral CD8 infiltration was evaluated using the chi-squared test. The ARID1A-negative group had significantly lower PD-L1 expression than the ARID1A-positive group. There was no significant difference in the expression of PD-1 and CD8 between the two groups (Table 2). In the univariable analysis, there was no significant difference in PFS or OS between the two groups (Figure 2).

### 3.3. Microsatellite Instability

**Figure 3:**
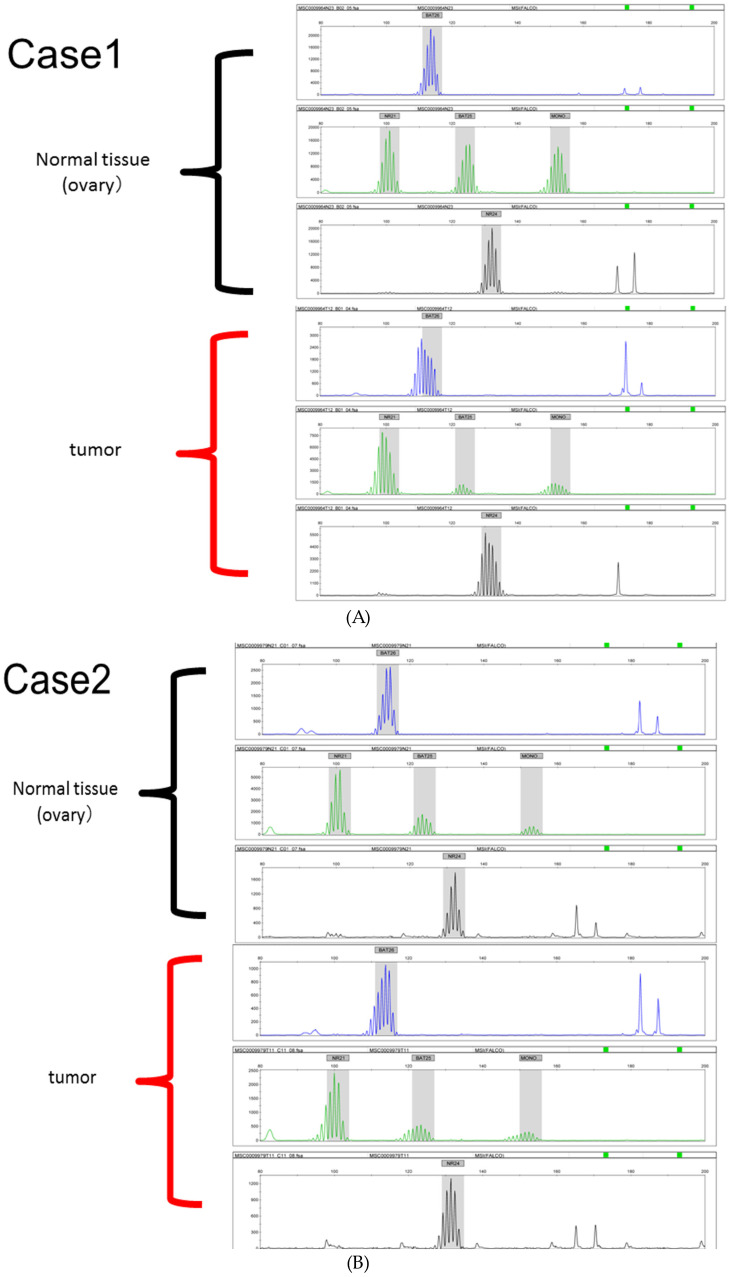

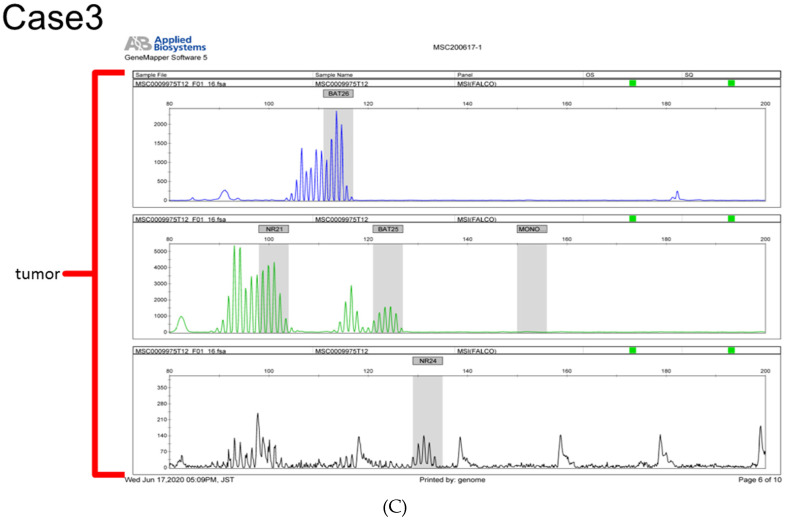
(**A**–**C**) Microsatellite instability (MSI) analysis of three endometrial carcinoma samples determined to be MSI-high.

MSI analysis was performed for the ARID1A-negative group. In total, 3 of the 21 samples (14.2%) were determined to be MSI-high (Figure 3).

### 3.4. Sanger Sequencing of ARID1A

**Figure 4:**
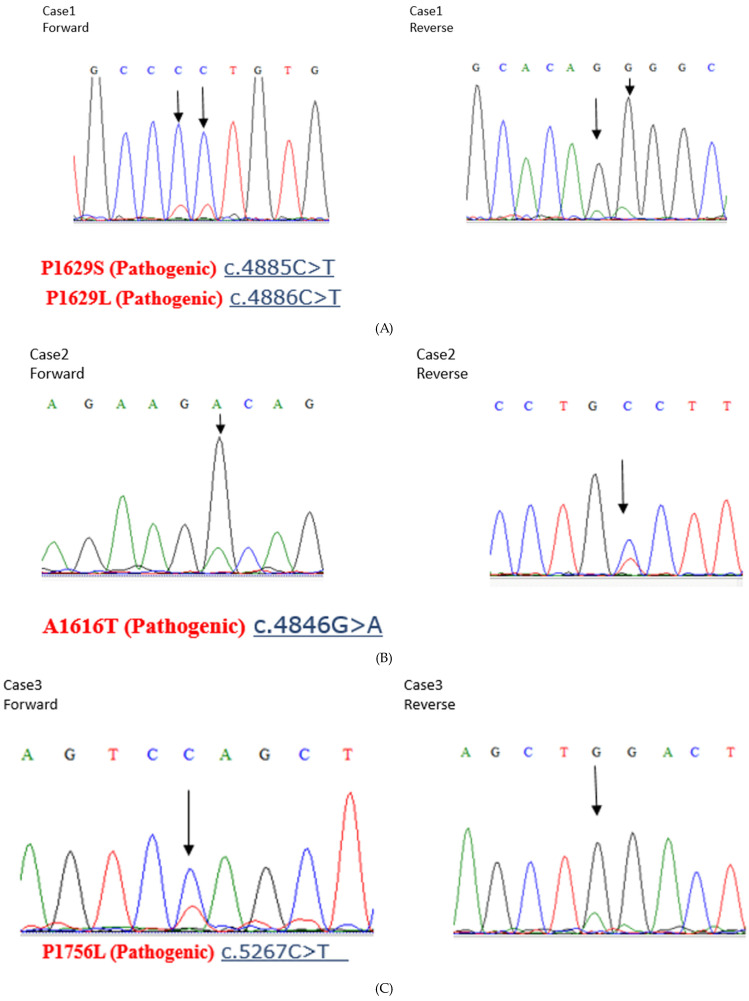

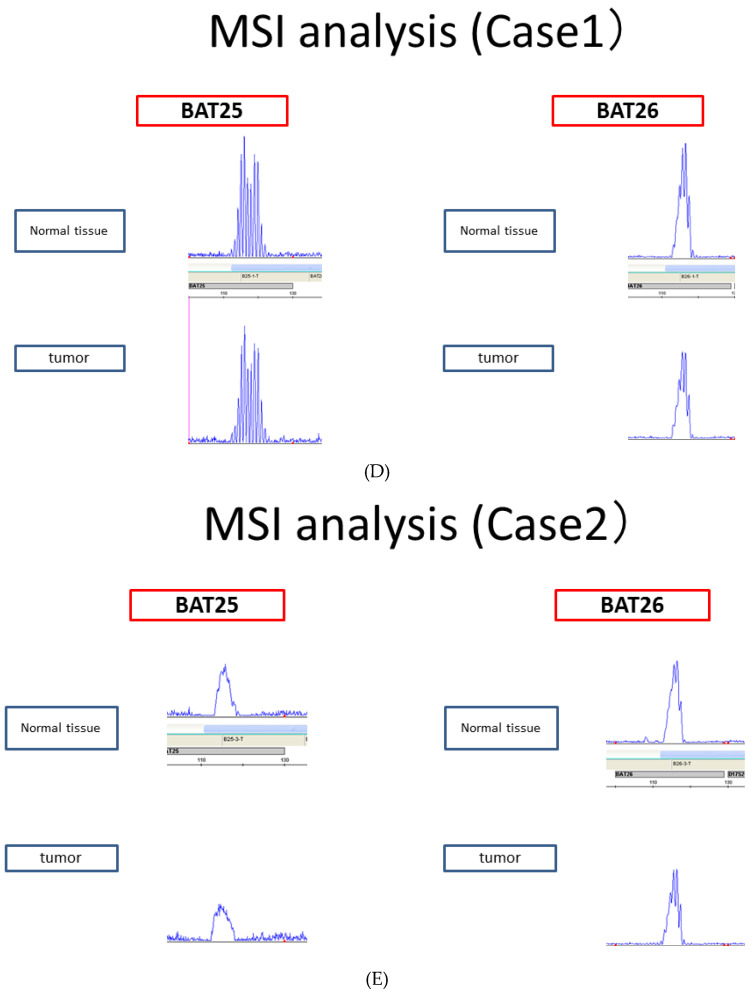

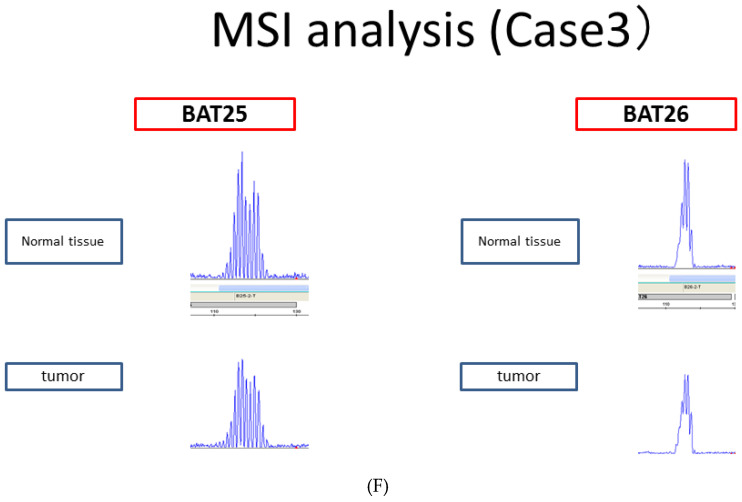
(**A**–**C**) Sanger sequencing of three endometrial carcinoma samples with pathogenic *ARID1A* mutations. (**D**–**F**). Microsatellite instability (MSI) analysis of the three endometrial carcinoma samples with pathogenic *ARID1A* mutations, showing negativity for all microsatellite markers.

The anti-ARID1A antibody used for immunostaining recognizes amino acids 600–1018 of ARID1A. As amino acids 1600–1800 of ARID1A interact with MSH2, the ARID1A-negative group identified through immunostaining cannot interact with MSH2. However, tumors with *ARID1A* mutations in the C-terminal side of the site recognized by the anti-ARID1A antibody show ARID1A expression through immunostaining but cannot interact with MSH2. Therefore, we performed the Sanger sequencing of *ARID1A* at the regions that bind to MSH2 (the region from amino acids 1600–1800 of ARID1A) in samples that were ARID1A-positive using immunohistochemistry. Of the 77 ARID1A-positive samples determined in this manner, 3 had pathogenic mutations (Figure 4A–C). However, the MSI analysis revealed that all three samples were MSS (Figure 4D–F). In addition, all three cases had low CD8 lymphocyte infiltration within the tumors.

### 3.5. MSI Analysis of HMOsis EC7

HMOsisEC7 ARID1A KO was negative for all microsatellite markers at both population doublings (PDs) 3 and 255 (Supplementary Figure S6).

## 4. Discussion

*ARID1A* mutations have been reported in various carcinomas. They are commonly found in gynecologic cancers, including ovarian cancer and endometrial cancer, and are present in 30–57% of tumors [19]. In this study, 24.5% of endometrial cancer samples were determined to be ARID1A-negative according to immunostaining, a result consistent with previous reports. Correlations between ARID1A expression and prognosis have been reported for various carcinomas. Several studies have found that ARID1A negativity is a poor prognostic factor [20,21,22,32,33,34], but other studies have found that it is a good prognostic factor [35,36]. In this study, there was no significant difference in PFS and OS between the ARID1A-negative and ARID1A-positive groups. Therefore, the relationship between ARID1A and prognosis is unclear. However, recurrent and advanced cancers are extremely resistant to chemotherapy and have poor prognoses. Therefore, it is thought that precision medicine employed according to genetic mutation is necessary. The discovery of new therapeutic strategies against ARID1A may improve the prognosis of endometrial cancer.

Shen et al. [14] reported that ARID1A interacts with MSH2 and that ICIs may be effective in tumors with *ARID1A* mutations. Since the publication of the report by Shen et al., *ARID1A* mutations have been reported to be biomarkers for ICIs in various cancers. However, most of the reports have not analyzed the expression of MMR proteins [24,37,38,39,40,41,42,43]. It has been reported that tumors with *ARID1A* mutations are significantly more likely than *ARID1A* wild-type tumors to have mutations of MMR genes [24]. A deficiency of MMR proteins may cause *ARID1A* mutations, thereby misleadingly indicating that *ARID1A* mutations are biomarkers for ICIs. There was no association between *ARID1A* mutations and OS in patients with MSS solid tumors in an ICI treatment cohort [42]. This result suggests that *ARID1A* alterations contribute to impaired MMR and mutator phenotypes in cancer. However, this contradicts the hypothesis that ARID1A interacts with MSH2. Therefore, it is unclear whether ARID1A is useful as a biomarker for predicting response to ICI therapy in patients with endometrial cancer.

To date, the association between ARID1A and immune-related molecules has been investigated only in relation to colorectal cancer, targeting MSS cases. Among the MSS colorectal cancers, tumors with *ARID1A* mutations showed higher expression of immune-related molecules (PD-1, cytotoxic T-lymphocyte antigen 4 (CTLA4)) and cytotoxic T cell infiltration compared to the *ARID1A* wild type [44]. Our study is the first report to investigate the relationship between ARID1A expression and immune-related molecules in MSS endometrial cancer. However, in this study, no correlation was found between ARID1A expression and CD8 lymphocyte expression in MSS endometrial cancer. It has been reported that high PD-L1 expression is a biomarker for ICIs [45,46,47]. However, the ARID1A-negative group had significantly lower PD-L1 expression than the ARID1A-positive group.

In addition, only three samples (14.2%) in the ARID1A-negative group were MSI-high. The Sanger sequencing of *ARID1A* in the region that binds to MSH2 detected pathogenic mutations in three samples. All three tumors were determined to be MSS according to MSI analysis. These results suggest that only a small proportion of patients with *ARID1A* mutations are likely to benefit from ICIs. Our results differ from those of previous studies on MSS colorectal cancers [44]. Therefore, whether ARID1A serves as a useful biomarker for predicting a patient’s response to ICI therapy may vary according to cancer type.

We subcultured HMOsisEC7 (ARID1A KO) and hypothesized that if ARID1A interacts with MSH2, the passage of HMOsisEC7 (ARID1A KO) would eventually result in an MSI-high status. However, contrary to expectations, HMosisEC7 (ARID1A KO) remained MSS even after passage to PDs255. Based on the results of this study, it is too early to consider ARID1A a biomarker for ICIs in endometrial cancer.

The main limitation of this study was its small sample size. Therefore, it is necessary to accumulate more cases and continue to examine whether ARID1A is unsuitable as a biomarker for ICIs. In addition, to evaluate immune cells, immunostaining was only performed for CD8 and PD-1. Recently, it has been reported that the production levels of cytokines such as interferon and interleukin-12 contribute to the efficacy of ICIs. Therefore, we believe that it is important to include these cytokine production levels in future studies [48,49,50].

## 5. Conclusions

In MSS endometrial carcinoma, no significant PD-L1 expression or immune cell infiltration were observed in the ARID1A-negative group. Thus, ARID1A is not suitable for use as a biomarker to predict response to ICI therapy among patients with endometrial cancer because there are few cases of MSI-high in patients with ARID1A-negative tumors. The results of this study are contrary to previous reports that ARID1A is useful as a biomarker for predicting the response to ICI therapy in other types of cancer. If *ARID1A* mutations are detected through genetic testing in patients with endometrial cancer in clinical practice, ICIs should be considered the recommended treatment, based on previous reports. However, as ARID1A was found to be unsuitable for use as a biomarker for deciding on ICI therapy for patients with endometrial carcinoma in this study, it is necessary to reconsider its use in genomic medicine.

## Figures and Tables

**Table 1:** Relationship between ARID1A and clinicopathological factors.

Characteristic	ARID1A Loss (n = 25)	ARID1A Positive (n = 77)	*p*-Value
Age-no. (%)			0.083
<60	8(32)	40(52)	
≥60	17(68)	37(48)	
Grade-no. (%)			0.741
G1	14(56)	45(58)	
G2,3	11(44)	32(42)	
FIGO Stage-no. (%)			0.544
I/II	18(72)	60(78)	
III/IV	7(28)	17(22)	
Pelvic lymph metastasis-no. (%)			0.598
No	18(90)	60(91)	
Yes	2(10)	6(9)	
Muscle invasion-no. (%)			0.619
<50%	15(63)	51(68)	
≥50%	9(37)	24(32)	

**Table 2:** Relationship between ARID1A and expression of PD-L1, PD-1, and CD8.

Parameter	ARID1A loss (n = 25)	ARID1A positive (n = 77)	*p*-Value
PD-L1: number (%)			0.017
Positive	2(8%)	25(32%)	
Negative	23(92%)	52(68%)	
Parameter	ARID1A loss (n = 25)	ARID1A positive (n = 77)	*p*-value
PD-1: number (%)			0.376
Positive	1(4%)	7(9%)	
Negative	24(96%)	70(91%)	
Parameter	ARID1A loss (n = 25)	ARID1A positive (n = 77)	*p*-value
CD8: number (%)			0.984
Positive	7(28%)	22(29%)	
Negative	18(72%)	55(71%)	

## Data Availability

The data presented in this study are available on request from the corresponding author.

## Supplementary Materials

The following supporting information can be downloaded at https://www.mdpi.com/article/10.3390/cancers16111999/s1. Figure S1: Cases studied; Figure S2: Representative images of ARID1A-positive and -negative cases; Figure S3: Relationship between anti ARID1A antibody used in the experiment and MSH2 binding site; Figure S4: Primer for exons 18-c, 18-d, 19, and 20a of ARID1A; Figure S5A: Original Images for Blots: ARID1A expression; Figure S5B: Original Images for Blots:GAPDH expression; Figure S6A: HMOsisEC7 ARID1A KO was negative for all microsatellite markers at PD 3; Figure S6B: HMOsisEC7 ARID1A KO was negative for all microsatellite markers at PD 255; Supplementary information: Plasmids.
