# Supplementary material for: Evaluation of ARID1A as a Potential Biomarker for Predicting Response to Immune Checkpoint Inhibitors in Patients with Endometrial Cancer
Source: Cancers (Basel). 2024 May 24;16(11):1999. doi: 10.3390/cancers16111999 (PMC11171230; doi:10.3390/cancers16111999)
Supplement: Supplementary file 1 [file cancers-16-01999-s001.zip › cancers-2994449-supplementary.pdf]

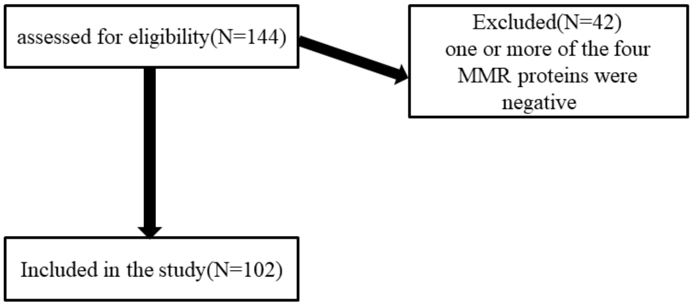


Supplementary Figure S1. We examined 102 cases of endometrial cancer with MMR protein expression.

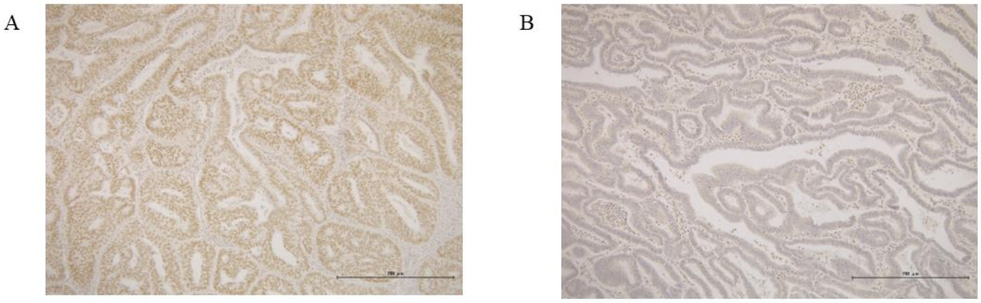


Supplementary Figure S2. Representative images of ARID1A positive and negative cases are shown.

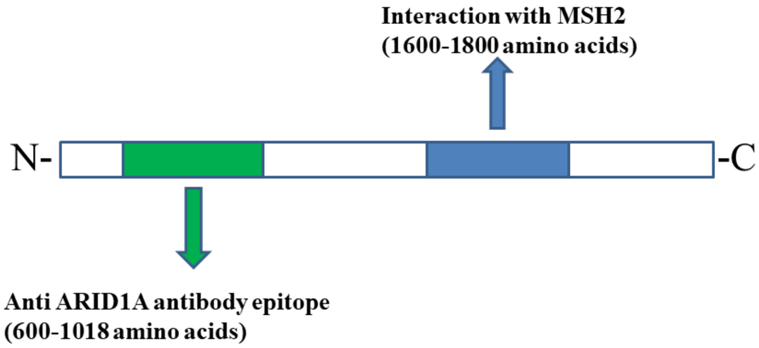


Supplementary Figure S3. An anti-ARID1A antibody that binds to 600-1018 amino acids was used for immunohistochemistry.

**ARID1A-18c**

Forward: GCTATGTGCGAGGCAGGTACT

Reverse: GCTCAGCAAGGCACCATGT

**ARID1A-18d**

Forward: ATTGCATGGCAATGAAGGAG

Reverse: CCTCCATCTAACTACCAGCCC

**ARID1A-19**

Forward: TGGCTAAAGATGAGACATTCCC

Reverse: AGACAGAAACTGCCTTCCACC

**ARID1A-20a**

Forward: GTCTTGCTCTCGAAGTGGGTC

Reverse: GGAGAACCTTTGGGAAAGGAG

Supplementary Figure S4. Primer for exon 18-c, 18-d, 19 and 20a of ARID1A

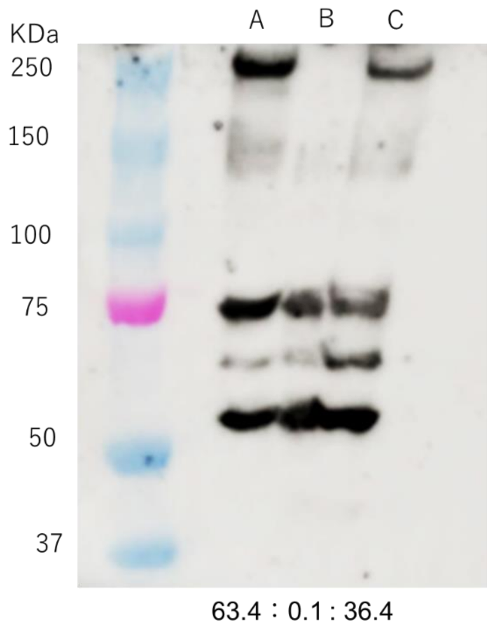


A = HMOsisEC7
B = HMOsisEC7 (ARID1A KO)
C = SKOV3

Supplementary Figure S5A. Original Images for Blots: ARID1A expression

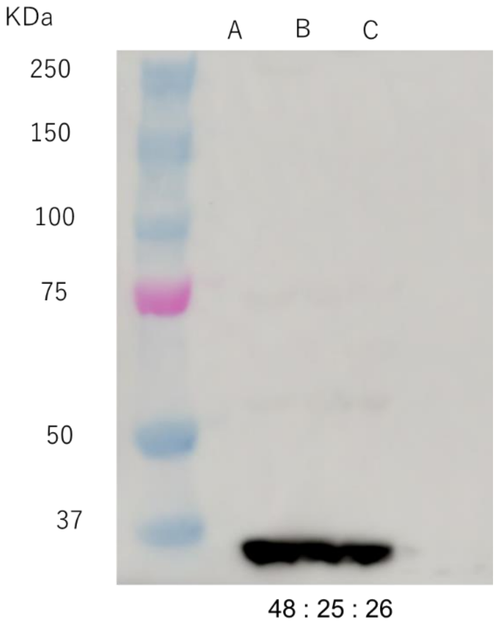


A = HMOsisEC7
B = HMOsisEC7 (ARID1A KO)
C = SKOV3

Supplementary Figure S5B. Original Images for Blots: GAPDH expression

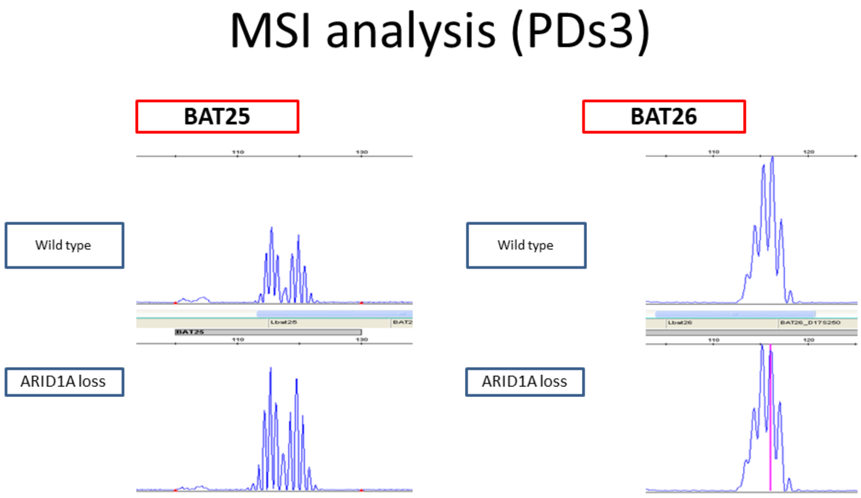


Supplementary Figure S6A. HMOsisEC7 ARID1A KO was negative for all microsatellite markers at PDs 3.

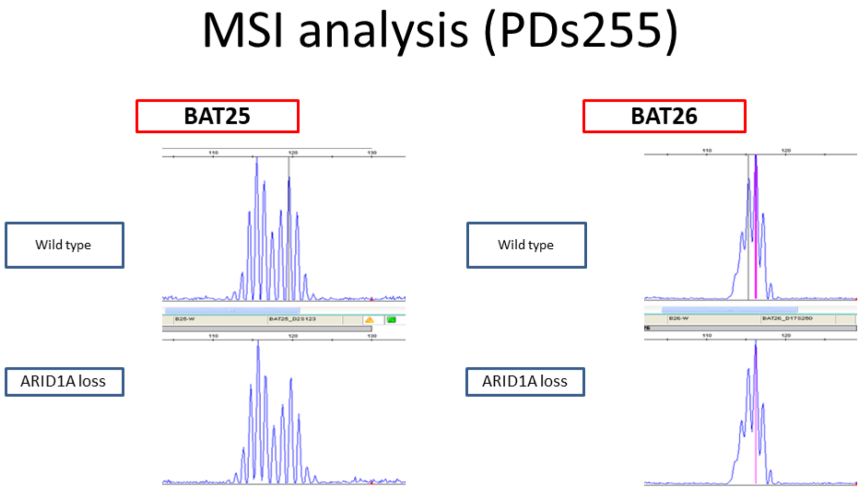


Supplementary Figure S6B. HMOsisEC7 ARID1A KO was negative for all microsatellite markers at PDs 255.

Supplementary information

## Plasmids

3xFlag-hCas9 segment from pX330-U6-Chimeric_BB-CBh-hSpCas9 was amplified by PCR with following primers, 5′-GGGGACAAGTTTGTACAAAAAAGCAGGCTgccaccATGGACTATAAGGACCAC-3′ and 5′-GGGGACCACTTTGTACAAGAAAGCTGGGTtaCTTTTTCTTTTTTGCCTGGC-3′, and recombined into pDONR221 to generate pENTR221-3xFlag-hCas9. pENTR221-3xFlag-hCas9 was recombined into PB-TAC-ERN by the LR recombinase (ThermoFisher Scientific) to generate PB-TAC-ERN-3xFlag-hCas9. pX330-U6-Chimeric_BB-CBh-hSpCas9 was a gift from Feng Zhang (Addgene plasmid # 42230 ; http://n2t.net/addgene:42230 ; RRID:Addgene_42230) (ref 1) and PB-TAC-ERN was a gift from Knut Woltjen (Addgene plasmid # 80475 ; http://n2t.net/addgene:80475 ; RRID:Addgene_80475) (ref 2).

Neomicin-resinstant gene in PB-TAC-ERN was swapped with puromycin-resistant gene by PCR and in-fusion reaction to generate PB-TAC-ERPuro. Tet-responsible promoter in PB-TAC-ERPuro was removed by PCR with primers, 5′-TCGAGTTAATTAACGAGAGCATAATATTGATATGTGCCAAAG-3′ and 5′-CGTTAATTAACTCGATCCGCGGTGGCGGCCATC-3′ and circularized by in-fusion reaction to generate PB-RfA-EPuro. EM7 promoter and puromycin-resistant gene in PB-RfA-EPuro was swapped with PGK promoter and HSV thymidine kinase (TK) fused with blasticidin-S-resistant gene (bsd) by in-fusion reaction to generate PB-RfA-PGK-TKbsd. pENTR221-U6H1R-gRNA_MCS contains U6 promoter, 5′-GAGGgcctatttcccatgattccttcatatttgcatatacgatacaaggctgttagagagataattagaattaatttgactgtaaacacaaagatattagtacaaaatacgtgacgtagaaagtaataatttcttgggtagtttgcagttttaaaattatgttttaaaatggactatcatatgcttaccgtaacttgaaagtatttcgatttcttggctttatatatcttg-3′ fused to 3′-end of H1 promoter sequence, 5′-atgagaccactctttcccg-3′ followed by multicloning site sequence 5′-AGATCTgggCTGCAGgggAAGCTT-3′ and gRNA scaffold sequence, 5′-gttttagagctagaaatagcaAGttaaaataaggctagtccgttatcaacttgaaaaagTGGCACCGAGTCGGTGC-3′ between attL2 and attL1 sequence of pENTR221. pENTR221-U6H1R-gRNA_MCS was amplified by PCR with primers, 5′-TATGGCGGGACTAACCCATcgggaaagagtggtctcat-3′ and 5′-ATGGGTTAGTCCCGCCATAgttttagagctagaaatagca-3′ and circularized by in-fusion

reaction to generate pENTR221-U6H1R-ARID1A-gRNA401-394. pENTR221-U6H1R-gRNA_MCS was amplified by PCR with primers, 5′-gtatggctgccctgggtaccgggaaagagtggtctcat-3′ and 5′-GTACCCAGGGCAGCCATACgttttagagctagaaatagca-3′ and circularized by in-fusion reaction to generate pENTR221-U6H1R-ARID1A-gRNA416-423. pENTR221-U6H1R-ARID1A-gRNA401-394 and pENTR221-U6H1R-ARID1A-gRNA416-423 were recombined with PB-RfA-PGK-TKbsd by LR reaction to generate PB-TKbsd-U6/H1R-ARID1A-gRNA401-394 and PB-TKbsd-U6/H1R-ARID1A-gRNA416-423, respectively. pCAG-PBase-M282V and pCAG-hyperPBase-i7EX (I30V/S103P/G165S/M282V/R372A/K375A/D450N/N538K/S509G/N570S)(ref 3) were constructed from pCAG-PBase by site-directed mutagenesis.

pX330-U6-Chimeric_BB-CBh-hSpCas9:

**ref 1. Multiplex Genome Engineering Using CRISPR/Cas Systems**. Cong L, Ran FA, Cox D, Lin S, Barretto R, Habib N, Hsu PD, Wu X, Jiang W, Marraffini LA, Zhang F. *Science. 2013 Jan 3.* 10.1126/science.1231143 PubMed 23287718


PB-TAC-ERN:

**Ref 2. Inducible Transgene Expression in Human iPS Cells Using Versatile All-in-One piggyBac Transposons**. Kim SI, Oceguera-Yanez F, Sakurai C, Nakagawa M, Yamanaka S, Woltjen K. *Methods Mol Biol. 2016;1357:111-31. doi: 10.1007/7651_2015_251.* 10.1007/7651_2015_251 PubMed 26025620


hyperPBase-i7EX

ref 3. Li X, Burnight ER, Cooney AL, Malani N, Brady T, Sander JD, Staber J, Wheelan SJ, Joung JK, McCray PB Jr, Bushman FD, Sinn PL, Craig NL. piggyBac transposase tools for genome engineering. Proc Natl Acad Sci U S A. 2013 Jun 18;110(25):E2279-87. doi: 10.1073/pnas.1305987110. Epub 2013 May 30. PMID: 23723351; PMCID: PMC3690869.
